# Phagocytosis of *Leptospira* by leukocytes from mice with different susceptibility to leptospirosis and possible role of chemokines

**DOI:** 10.1186/s12866-018-1371-9

**Published:** 2019-01-07

**Authors:** Paloma L. da Silva, Fabiana Lauretti-Ferreira, Maiara Caldas de Lima, Swiany Silveira Lima, Ambart E. Covarrubias, Marcelo De Franco, Eneas Carvalho, Paulo Lee Ho, Renata M. A. da Costa, Elizabeth A. L. Martins, Josefa B. Da Silva

**Affiliations:** 1 Laboratório de Bacteriologia, Instituto Butantan, São Paulo, Brazil; 2 Fundação Ezequiel Dias, Belo Horizonte, Brazil; 3 School of Medical Technology, Faculty of Health Sciences, University San Sebastian, Concepción, Chile; 4 Instituto Pasteur, São Paulo, Brazil; 5 Seção de Vacinas Aeróbicas, Instituto Butantan, São Paulo, Brazil; 6 Laboratório de desenvolvimento de processos, Instituto Butantan, São Paulo, Brazil; 7 Present address: Global Antibiotics Research and Development Partnership (GARDP), Drugs for Neglected Diseases initiative (DNDi), Chemin Louis-Dunant 15, 1202 Geneva, Switzerland

**Keywords:** Phagocytosis, *Leptospira*, Chemokines, CCL2/MCP-1, C3H/HeJ, C3H/HePas

## Abstract

**Background:**

Leptospirosis is a widespread zoonosis caused by pathogenic prokaryotic microbes of the genus *Leptospira*. Although there are several reports in the literature, host-pathogen interaction is still poorly understood. The role of chemokine expression is important on the chemotaxis, activation and regulation of immune cells. Recent studies have shown that their expression profiles play an important role on the severity of leptospirosis outcome. We evaluated the phagocytosis of *Leptospira* by spleens cells from C3H/HeJ, C3H/HePas and BALB/c mouse strains, respectively susceptible, intermediate and resistant to leptospirosis, and by RAW 264.7 macrophages. Besides, we evaluated the effects of CCL2 treatment on the phagocytosis. The cells were incubated with or without CCL2 chemokine, and infected with virulent *L. interrogans* sv Copenhageni. Cells and culture supernatants were collected for subsequent analysis.

**Results:**

The number of leptospires was higher in BALB/c cells, CCL2 pre-treated or only infected groups, when compared to C3H/HeJ and C3H/HePas cells. Indeed, CCL2 activation did not interfere in the phagocytosis of *Leptospira*. Expression of chemokines CXCL5 and CCL8 levels were significantly inhibited in infected BALB/c cells when compared to the non-infected control.

**Conclusions:**

Higher ability to phagocytosis and early modulation of some chemokines correlated with the resistance to leptospirosis disease. Exposure to CCL2 did not interfere on phagocytosis of *Leptospira* in our experimental conditions, but acted in the modulation of chemokines expression during *Leptospira* infection.

**Electronic supplementary material:**

The online version of this article (10.1186/s12866-018-1371-9) contains supplementary material, which is available to authorized users.

## Background

Leptospirosis is an emerging zoonosis, caused by pathogenic species of the genus *Leptospira* that affects several animals and humans. *Leptospira* spp. have the ability to colonize renal tubules in reservoir hosts, usually rodents, which excrete the leptospires in their urine, contaminating the environment. Infection in humans can occur through contact of skin or mucous membrane abrasions with contaminated either water or moist soil. The clinical manifestations are very heterogeneous, from mild flu-like to more severe cases such as leptospirosis-associated pulmonary hemorrhagic syndrome and Weil’s Disease [1, 2].

Leptospirosis is one of the main zoonosis that causes human morbidity and mortality, with estimates of about one million cases and 58,900 deaths per year worldwide [3]. Host-pathogen relationship in leptospirosis is still poorly understood. Considering the strong impact of this zoonosis on public health, investigation is necessary to subsidize strategies for control and prevention of the disease.

Hamsters are the rodent model most used for studies of leptospirosis, because these animals are susceptible to acute infection. Mice in general are asymptomatic reservoirs, however studies have shown that young mice from C3H/HeJ strain, deficient to Toll-like receptor 4 (TLR4), are susceptible to infection by *Leptospira* and represent an ideal model for immune response studies [4–6].

The innate immune system protects the host from pathogens through recognition, recruiting of immune cells to the infected sites and activation of the adaptive immune response [7]. TLRs are involved in these processes. Most bacteria are recognized mainly by TLR4 mediated by lipopolysaccharides (LPS) present in its outer membrane, contributing to the activation of immune response. Recovery from the foreigner stimulus depends on a robust yet tightly regulated innate and adaptative immune responses [8, 9].

The immunologic consequences of phagocytosis vary depending on the cell type, the receptors involved in recognition and uptake, and the nature of the infection. Macrophages contain a diversity of molecules, such as acid hydrolases that extensively degrade ingested macromolecules. Besides, the phagocytosis of foreign molecules, microorganisms, apoptotic and necrotic cells result in anti- and pro-inflammatory consequences to the host [10]. Thus, strong regulation of macrophage activation mediated by chemokine/cytokine is central for an adequate immunity, as improper activation of the macrophages can lead to immunopathology [11].

Dendritic cells (DCs) are also phagocytic and professional antigen-presenting cells, participating in the activation of specific T-cell. The localization of DCs is strictly regulated by a large variety of chemotactic and nonchemotactic signals, generating a complex regulatory network through synergistic interactions, proteolytic processing, and actions of chemokines and atypical chemokine receptors [12].

Chemokines are chemotactic cytokines, mostly expressed by leukocytes, which mediate the innate immune system responses, recruiting leukocytes to the site of infection. However, exacerbated inflammation induces tissue damage and the maintenance of the inflammation can lead to detrimental effects on the host [13].

On the other hand, proteolytic cleavage of some chemokine, such as CCL8, devoid the chemotactic activity on monocyte, reducing the inflammation [14, 15].

CCL8 (monocyte chemotactic protein-2, MCP-2), from CC chemokine sub-family, has been reported as an agonist of C-C chemokine receptor type 2 (CCR2) and CCR5, and plays a pivotal role in the control of leukocyte chemotaxis [16].

CXCL5 has a critical role recruiting and controlling neutrophils traffic in response to bacterial infections. It was reported the participation of this chemokine in lipopolysaccharide (LPS)-induced lung inflammation in mice and also that the inhibition of CXCL5 expression results in exaggerated neutrophil-mediated inflammation in pulmonary bronchiolar cells [17, 18], illustrating the modulating role of the chemokine.

Chemokines feature an important role as pro-inflammatory signaling proteins, as consequence of their chemoattractant and its direct antimicrobial properties [19]. These activities seem to be related to the high positive net charge of these immune proteins [20].

Bactericidal activities of thirty chemokines were evaluated and eighteen of them harbored activities against *Escherichia coli* and *Staphylococcus aureus* [21]. The antimicrobial activities were further evaluated and confirmed for other chemokines [22–24].

CCL2/MCP-1 (monocyte chemotactic protein 1) is one of the most studied molecules among chemokines. This mediator belongs to the CC family and acts as a chemotaxis factor for monocytes/macrophages, NK cells and memory T lymphocytes. CCL2/MCP1 is produced by a variety of cells, including monocytes/macrophages, endothelial cells, fibroblasts and epithelial cells. It has high affinity to its CCR2 receptor, which is expressed by several types of leukocytes. Expression of CCL2/CCR2 has been correlated to different pathological conditions, such as rheumatoid arthritis, atherosclerosis and multiple sclerosis [25, 26].

In chronic pulmonary diseases, macrophages release high levels of pro-inflammatory cytokines and chemokines, including CCL2, driving the recruitment of other inflammatory cells, including neutrophils and monocytes, to the lungs, promoting the disease progression [27].

Our previous studies showed that *Leptospira* induces changes in the levels of chemokines in organs of mice in the first hours after infection, including CCL2/MCP-1 [28]. However, it is not yet clear if this phenomenon contributes for *Leptospira* escaping from the immune system’s actions, or if it correlates to the resistance / susceptibility phenotypes of the mouse strains to leptospirosis.

In order to investigate a possible correlation of resistance to leptospirosis and leukocytes’ phagocytosis we evaluated the capacity of spleen cells isolated from C3H/HeJ (toll-like receptor 4 deficient), C3H/HePas and BALB/c mouse strains and the RAW 264.7 macrophages to phagocyte *L. interrogans* serovar Copenhageni, and possible effects of CCL2/MCP-1 treatment.

We also analyzed the induction of the chemokines CCL5/RANTES, CCL12 CXCL10/IP-10, and CXCL12/SDF-1, which are strongly related to regulation of lymphocyte migration, CCL8/MCP-2, a monocytes chemoattractant, CCL9/MIP-1γ that regulates different immune process and chemokine signaling pathways, and CXCL5/LIX that participates in the recruitment of inflammatory cells in case of injured or infected tissues.

## Materials and methods

### Culture of *Leptospira* and maintenance of virulence

In this study *L. interrogans* serovar Copenhageni strain (ATCC® BAA-1198™) was used, and its virulence was maintained by successive passages in golden hamster (*Mesocricetus auratus*) as previously described [29]. Leptospires were recovered from the organs of infected animals and cultured in Ellinghausen-McCullough-Johnson-Harris (EMJH) medium [30], at 30 °C under aerobic conditions. After 4 to 6 days of culture growth, the number of leptospires was counted in a Petroff-Hausser chamber and the suspension was used for in vitro infection of RAW 264.7 (ATCC-TIB-71 provided by the Banco de Células do Rio de Janeiro-BCRJ). Macrophage and spleen cells from three mice strains, which display different susceptibilities to leptospirosis: susceptible (C3H/HeJ), intermediate (C3H/HePas) and resistant (BALB/c) mouse strains. The animals were provided by the Immunology Department of Institute of Biomedical Sciences of University of São Paulo, Brazil.

### Mice spleen cells processing and RAW 264.7 cells

Six spleens were aseptically collected (3 spleens from each strain in two consecutive experiments). For collection of spleens, mice were euthanized through the ip. (intraperitoneal) route with a lethal dose of a xylazine/ketamine solution (60 mg/Kg of xylazine and 300 mg/Kg of ketamine).

Spleens were macerated and suspended in RPMI supplemented with 10% heat-inactivated bovine fetal serum, 10 μg of streptomycin and 1% L-glutamine (complete RPMI). Erythrocytes were lysed with ACK Lysing Buffer (Gibco™) and the remaining cells were collected by centrifugation. The cells were washed twice with RPMI without antibiotic. Resulting cells were counted in a Neubauer chamber, and then placed in 6-well plates for culture with a concentration of 1 × 10^6^ cells/well. Cells were treated with CCL2 chemokine and infected. The RAW 264.7 cells were also cultured in the same medium, following appropriate culture conditions [31].

### Experimental infection of cells with virulent *L. interrogans* sv Copenhageni

Two groups of cells were established, one for treatment with 1 ng/ml of recombinant mouse CCL2/MCP-1 (R&D Systems) and other, non-treated, as the control group. Cells were maintained in RPMI medium without serum or antibiotics. After 30 min, treated and non-treated cells were infected with virulent *L. interrogans* sv Copenhageni (1 × 10^7^) in a multiplicity of infection (MOI) equals 10 for 1 h. Next, the supernatants were collected and cells were washed twice with RPMI with antibiotics and then incubated in RPMI with antibiotics. After 1 h, the cells were washed and recovered for analysis and immediately stored at − 80 °C. The RAW cells were also processed for confocal microscopy analyses.

### DNA extraction and quantitative PCR (qPCR)

The number of retained leptospires was determined by qPCR after extraction of total genomic DNA from infected cells. The total genomic DNA was extracted using a DNA extraction kit (DNeasy tissue - Qiagen), according to the manufacturer’s instructions. DNA samples were quantified in a NanoDrop 1000 spectrophotometer (Applied Biosystems). The oligonucleotide sequences used in this study refer to *Leptospira* gene *16 s* rRNA: primer foward 5’-TTCAGTTGGGCACTCGTAAG-3′ and primer reverse 5’-CGTGTGTTGCCCTAGACATAA-3′. The qPCR reactions were carried out using Syber Green Master Mix (Applied Biosystems, USA) in a 12 μl final volume, containing 1 ng of DNA (extracted from each cell culture sample or from L*eptospira* cultured for 5 days at 30 °C in EMJH medium, as a positive control), and 4 μmol of each primer (forward and reverse). The qPCR reactions were performed and analyzed using the Applied Biosystems 7300 Real-Time PCR System. PCR efficiency was determined for each individual reaction, using the software LinRegPCR [32]. All oligonucleotides had the correlation coefficient squared (R2) higher or equal to 0.998 and the range of the efficiency was 1.9–2.0. All reactions were close to 100%, which is indicative of a stable and reliable assay. All qPCRs were performed in triplicate and the results represent the data of two individual experiments. Triplicates of 10-fold serial dilutions (10^− 1^ to 10^− 8^) of 100 pg initial concentration of genomic DNA from *L. interrogans* sv Copenhageni were used to construct a standard curve for determination of the number of phagocytosed leptospires. The initial sample of cultured contained 3.7 × 10^8^ leptospires. The last dilution that could amplify the target at threshold cycle (Ct 34) was selected as the Lower Limit of Detection. Data of Ct value at different concentrations of DNA were submitted to regression analyses and the equation was used to calculate the phagocytosed leptospires in different treatments.

### Immuofluorescence and confocal microscopy

Following the infection of cells with *Leptospira*, samples were washed with PBS and fixed with 2% paraformaldehyde for 10 min at room temperature. Subsequently, cells were permeabilized by 1% Triton X-100, allowing antibodies to penetrate them for immunofluorescent staining. Purified antibodies anti-*L. interrogans* made in rabbit were used as primary antibodies, gently supplied by Dr. Vasconcellos S. (Universidade de São Paulo). Antibody anti-rabbit IgG FITC-conjugated (Alexa fluor 488-goat Anti-Rabbit IgG (H + L) Life Technology) was used as secondary antibody. Evans blue dye (Sigma) was used to stain the cell cytoplasm and DAPI to stain the nucleus of cell. The samples were observed under a confocal microscope (Leica TCS SP8 - Germany). The images were acquired at different cross-sections, analyzed by 3D and orthogonal tools, which respectively provide a live rotation and cross-sections of any localization, allowing for the visualization and quantification of phagocytosed leptospires**.**

### Chemokines determination by ELISA and protein interaction by data base analysis

Supernatants from cell cultures were used for determination of the chemokines: CCL5/RANTES, CCL8/MCP-2, CCL9/MIP-1γ, CCL12, CXCL5/LIX, CXCL10/IP-10, and CXCL12/SDF-1 expression levels by ELISA using commercial kits (R&D systems). The protein-protein interaction networks of CXCL5 and CCL8 were analyzed by GeneCard-GPS data base, These chemokines were chosen because they presented statistical significance differences in our study.

### Statistical analysis

One-way ANOVA and Turkey post-multiple comparison tests were applied to assess significant differences (*p-values*) on numbers of phagocytosed leptospires and chemokine expression in samples analyzed in the different treatments of cells. For statistical analysis, any values outside one absolute deviation around the median (MAD-median method) were considered outlier and discarded. Statistical analysis and plotting of data were performed using Prism software (GraphPad).

## Results

### Phagocytosis of *Leptospira*

To evaluate the possible effects of chemokines on phagocytosis of *L. interrogans*, spleen cells from three mice strains C3H/HeJ, C3H/HePas and BALB/c with different susceptibility phenotypes, as well as RAW 264.7 macrophages were evaluated. Cultured cells were treated with chemokine CCL2/MCP-1 and subsequently the cultures of treated and non-treated cells were infected with *L. interrogans* sv Copenhageni. The number of phagocytosed *Leptospira* and the expression levels of other chemokines were analyzed.

**Fig. 1:**
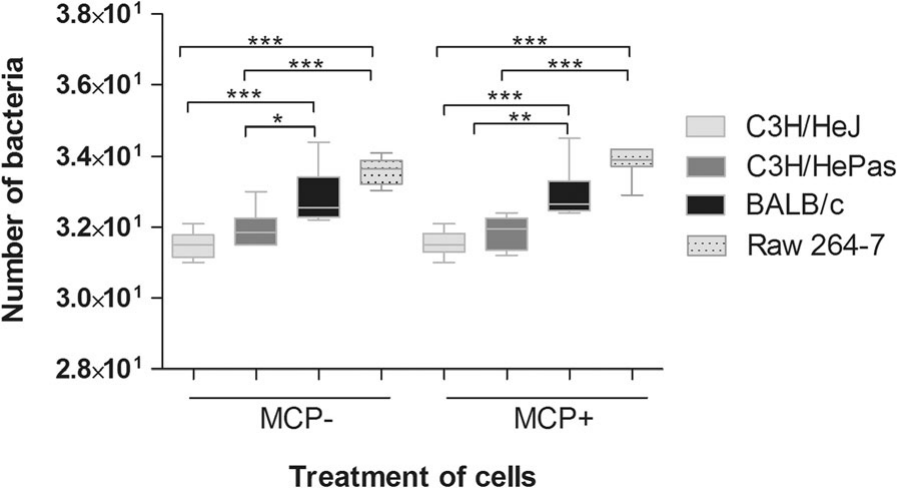
Number of *L. interrogans* in spleen cells from C3H/HeJ, C3H/HePas and BALB/c mouse strains, and Raw 264.7 macrophages treated with CCL2/MCP-1 (MCP^+^) or untreated (MCP^−^) chemokine and analyzed 2 h after infection by qPCR. Statistical differences and multiple comparison analysis were performed (*p < 0.05; **p < 0.001; ***p < 0.0001). Data are mean ± SD, *n* = 6

The number of retained leptospires on the cells isolated from Balb/c mice was higher than on cells from C3H/HeJ or C3H/HePas mice (Fig. 1) as analyzed by qPCR, confirming a correlation between phagocytosis capacity and resistance to leptospirosis. In our experimental conditions, RAW 264.7 macrophages displayed the highest efficiency to retain *Leptospira* when compared to mice spleen cells. CCL2 treatment did not interfere on the number of retained leptospires in any of the cells analyzed (Fig. 1).

**Fig. 2:**
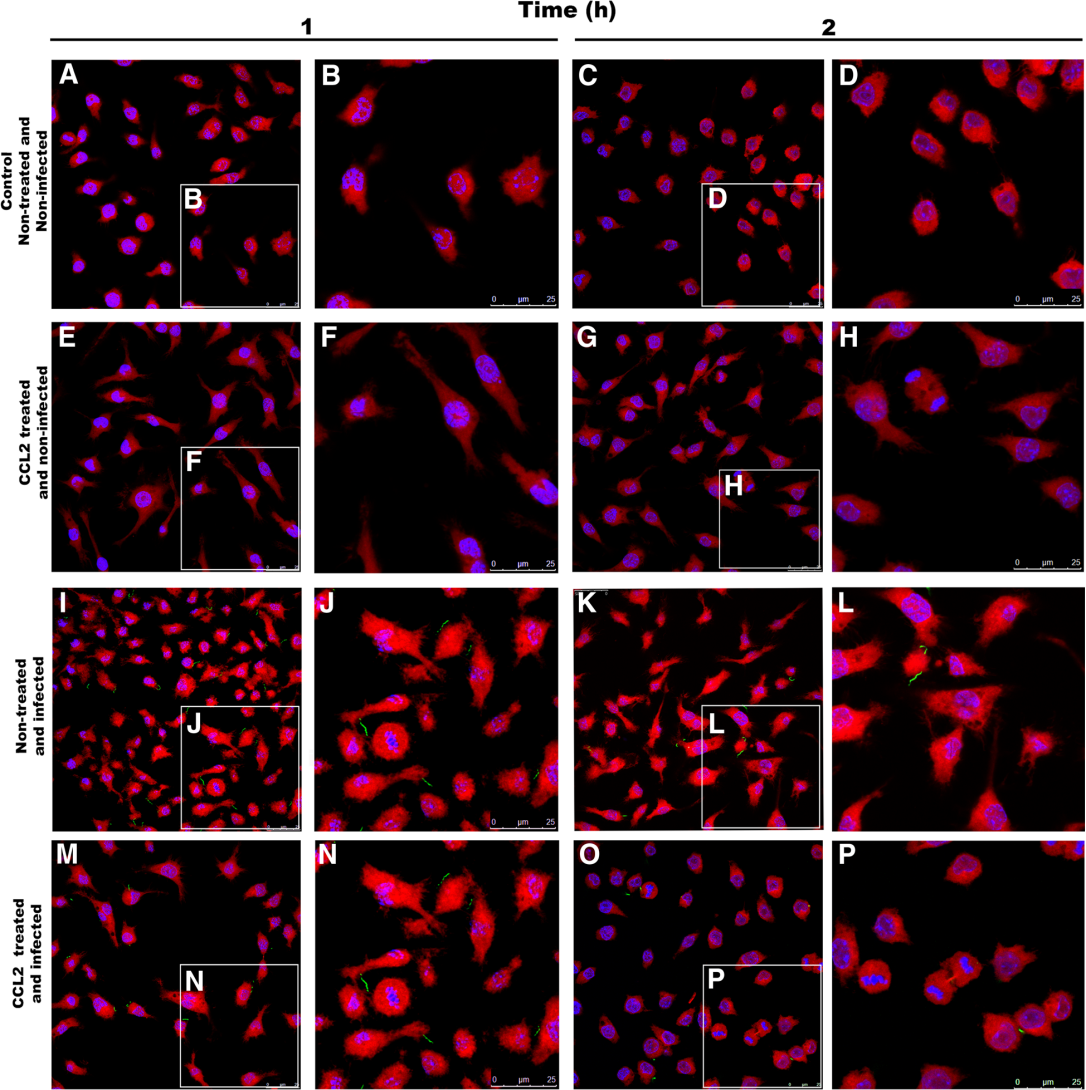
Images of RAW 264.7 macrophages treated with CCL2 chemokine and infected by *L. interrogans,* analyzed by confocal microscopy 1 h or 2 h after infection. **a** and **c**) Control cells, non-treated and non-infected; **e** and **g**) Cells CCL2-treated and non infected; **i** and **k**) Cells CCL2 non-treated and infected; **m** and **o**) Cells CCL2-treated and infected. *L. interrogans* detected in green by antibodies FITC-conjugated. Nucleus of cells stained in blue by DAPI marker, cell’s cytoplasm stained in red by Evans blue. Amplifications of slides (**b**, **d**, **f**, **h**, **j**, **l**, **n** and **p**) are indicated in white squares

**Fig. 3:**
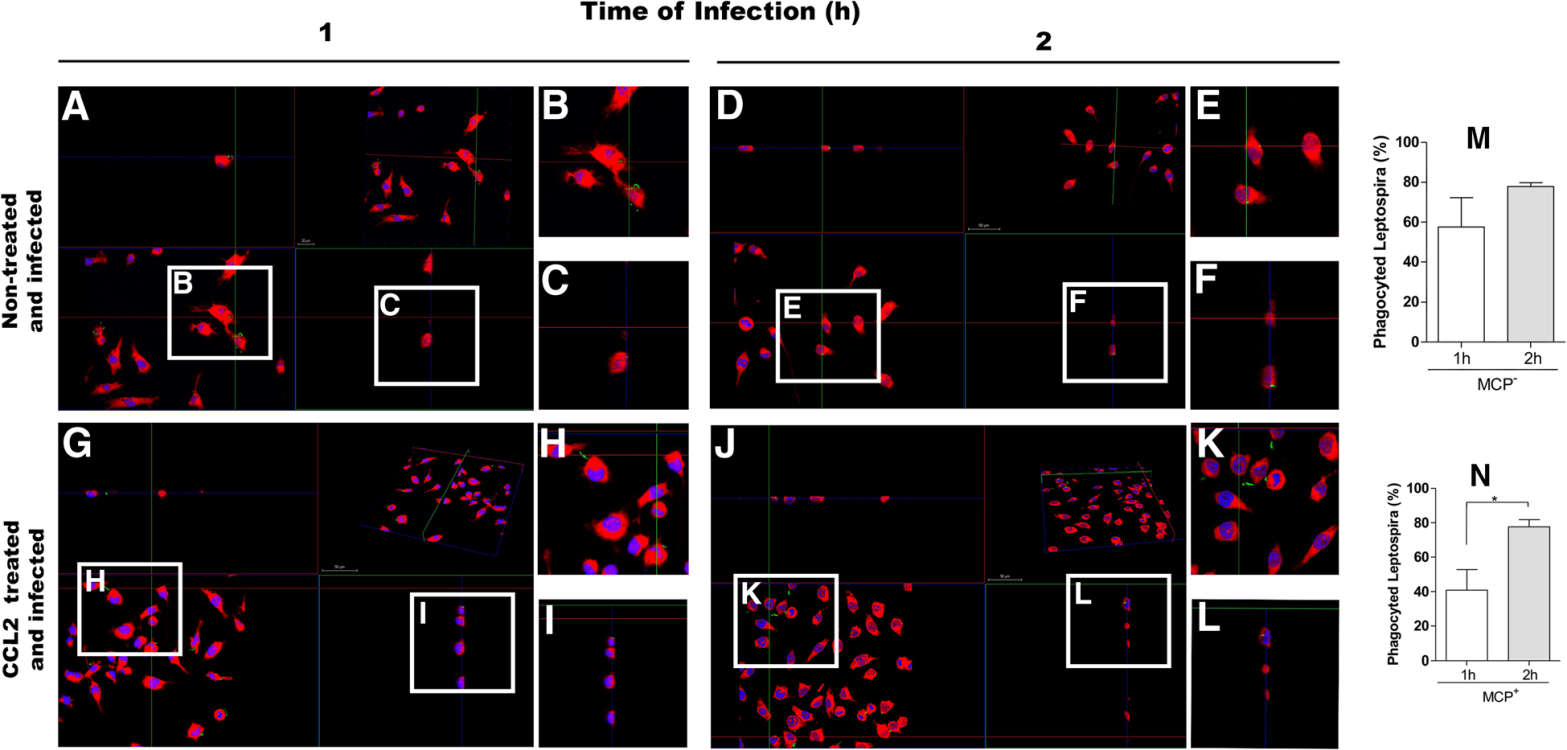
Orthogonal images of RAW 264.7 macrophage CCL2-treated and non-treated, and infected by *L. interrogans*, allowing the observation of phagocytosed leptospires in different planes indicated in white squares. **a** and **d**) infected cells analyzed 1 h and 2 h post-infection, respectively; **g** and **j**) Cells CCL2-treated and infected, analyzed 1 h and 2 h post-infection respectively. White squares **b**, **c**, **e**, **f**, **h**, **i**, **k** and **l** on the images  are presented also amplified. **m** and **n**- percentage of pagocyted leptospires in the total of detected, by CCL2 non treated or treated cells respectively, in 1 h or 2 h after the infection. *L. interrogans* detected by antibodies FITC-conjugated. Nucleus of cells stained in blue by DAPI marker, cell’s cytoplasm stained in red by Evans blue. Leptospires were counted over five fields of each slide, being prepared three slides from each experimental treatment. Data are mean ± SD. Statistical differences and multiple comparison analysis were performed (*p < 0.05)

To confirm the internalization of *Leptospira* in the RAW 264.7 cells, we used indirect immunofluorescence and images were visualized by confocal microscopy (Figs. 2, 3). It was possible to confirm the phagocytosis of *L. interrogans* at 1 h and 2 h post-infection (Fig. 3). The number of phagocytosed leptospires was documented in the 3D confocal images and analyzed by ortho images counting (Fig. 3), allowing the observation of phagocytosed leptospires in several cellular planes. Leptospires bound to the cell’s membrane or in the intercellular spaces were observed in the different times of analysis (Fig. 2-3). The proportion of phagocyted versus extracellular leptospires was plotted (Fig. 3).

There was no statistical difference between the number of phagocytosed leptospires in CCL2 treated and non-treated cells from the same mouse strains, as measured by qPCR analysis (Fig. 1), indicating that there was no interference of this chemokine in the phagocytosis in our experimental conditions. However, statically significant difference in leptospires phagocytosis by RAW cells CCL2-treated was observed between 1 h and 2 h after infection (Fig. 3 M-N).

### Expression of chemokines after CCL2 treatment of cells from mice with different phenotypes of resistance to leptospirosis

**Fig. 4:**
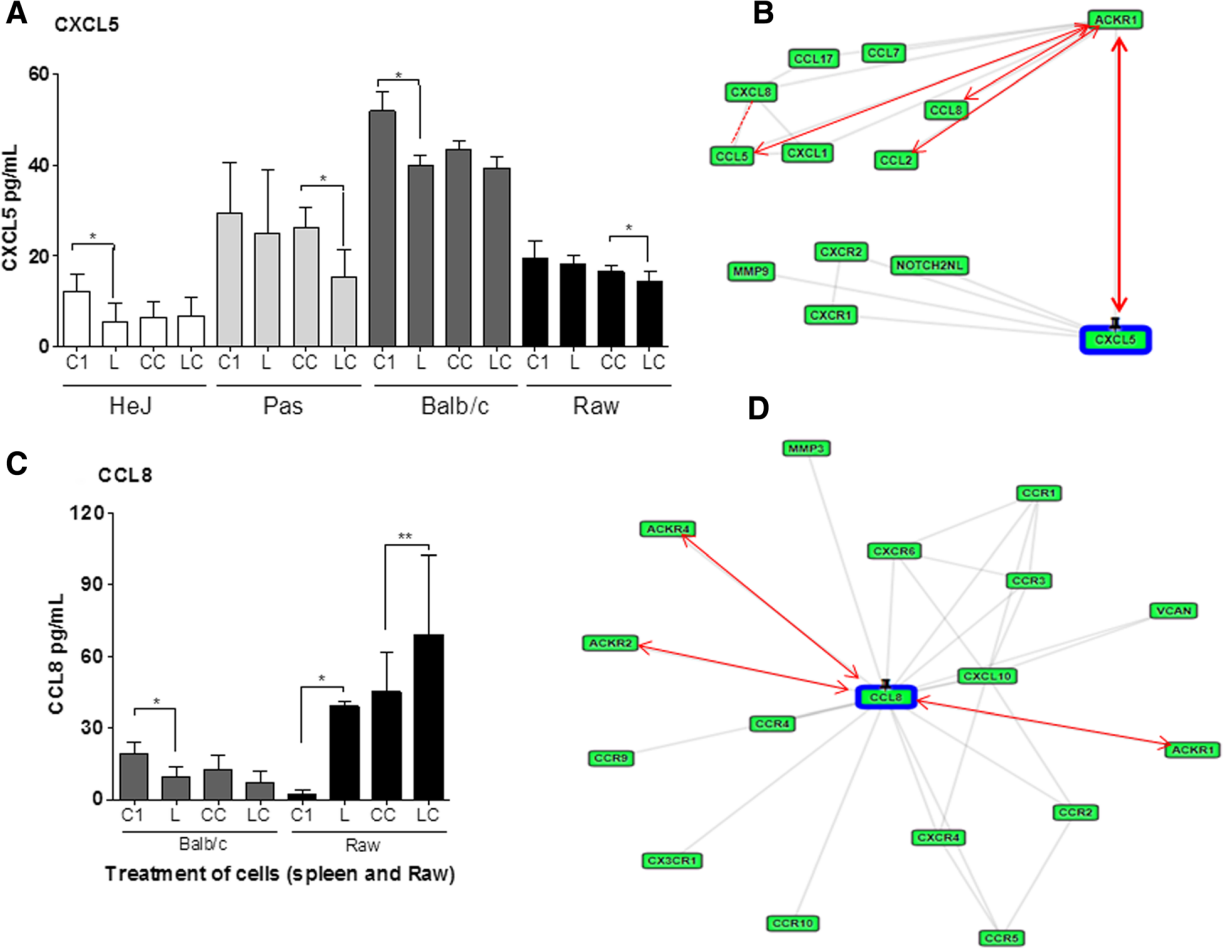
Chemokines in spleen cells from three mouse strains and in RAW 264.7 cells, 1 h after the infection, measured by ELISA. **a** and **c**) CXCL5 and CCL8 levels respectively: C1) control; CC) Control CCL2-treated; L) leptospires infected cells; LC) leptospires infected and CCL2-treated cells. Data are mean ± SD (n = 6). Statistically significant differences and multiple comparison analysis were calculated (*p < 0.05; **p < 0.001). **b** and **d**) Respectively CXCL5 and CCL8 protein-protein interaction network analysis, by GeneCard-GPS database. Red arrows represent the direct and dashed lines indirect interaction

**Fig. 5:**
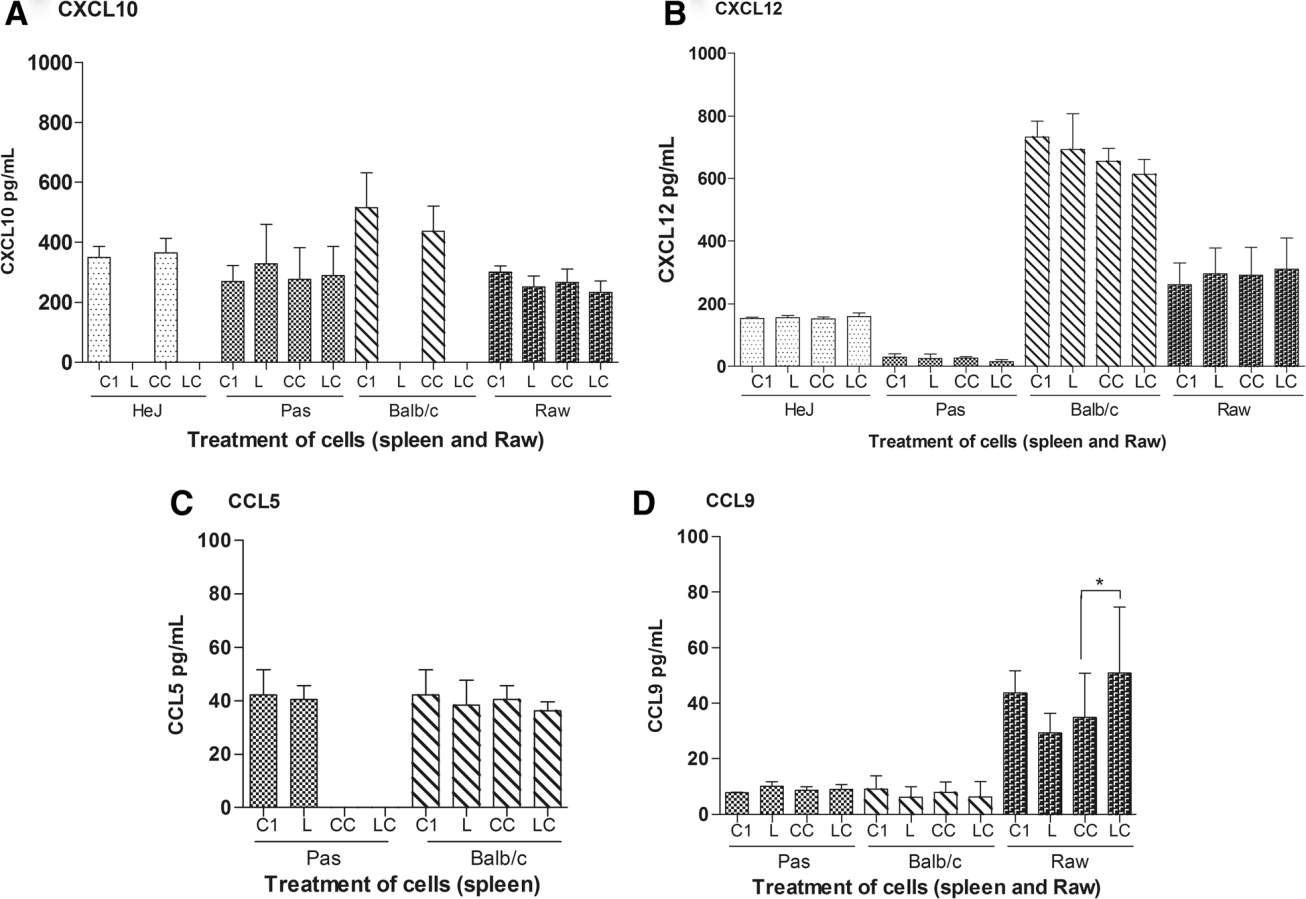
Chemokines CXCL10, CXCL12, CCL5 and CCL9 (panels **a**, **b**, **c** and **d**) in spleen cells from three mice strains and RAW 264.7 cells, measured by ELISA, 1 h after the infection. C1 = control; CC = control CCL2 treatment; L = leptospires; LC = leptospires and CCL2 treatment. Data are mean ± SD, *n* = 5). Statisticaly significant differences and multiple comparison analysis were performed (*p < 0.05)

Chemokine profiles of spleen cells from the three mice strains after 1 h post infection were evaluated by ELISA. The levels of CXCL5 were reduced in infected cell from the three mice strains (Fig.4, 5), however, in cells from C3H/HePas and in RAW macrophages the reduction occurred only after CCL2 treatment (Fig. 4A). The CCL8 chemokine was expressed only by RAW macrophages and BALB/c spleen cells. After leptospires infection CCL8 expression was reduced in BALB/c cells, while increased in RAW cells (Fig. 4B). The findings suggest a possible role of CXCL5 and CCL8 molecules.

CXCL5 and CCL8 molecules interact with several highly bioactive molecules, participating in different protein interaction cascades, as shown by data base analysis (Fig. 4 M-N). The profile of these chemokines as observed in our analysis may suggest two opposite effects: a) an action to control the exacerbated immune response or, b) they could induce a dissemination of leptospires in the host. The strategy of *Leptospira* dissemination in the host, was supposed since these chemokines were inhibited in the resistant strain and was not detected in cells isolated from susceptible strains to leptospirosis.

For the remaining chemokines, CXCL10, CXCL12, CCL5 and CCL9, there was no significant difference of expression. Only CCL9 had lower expression in RAW infected cells as we showing (Fig. 5).

## Discussion

Lethal infection of mice depends on strain, age, and *Leptospira* serovars. In general, mice and rats behave as reservoir hosts of the bacterium and present resistance phenotype to infection [1]. However, mice deficient in specific molecules of the immune system, such as C3H/HeJ mice (tlr4^−^/^−^), are highly susceptible to lethal infection with *L. interrogans* serovar Icterohaemorrhagiae and Copenhageni [6, 29, 33, 34].

Few virulence factors have been described in *Leptospira* including lipoproteins [35], proteins involved with motility [36], and essential molecules for the uptake of iron [37], and its survival capacity within macrophages [38, 39].

This investigation was conducted to understand if the phenotype differences of mouse strains contribute for reducing the number of leptospires and CCL2 contributes to the phagocytosis process of *L. interrogans* by mice spleen cells and RAW cells in vitro. Our results showed that the rate of leptospires correlates with the resistance to leptospirosis as the number of leptospires detected in cells from the resistant strain BALB/c was higher than the ones from susceptible strains (C3H/HeJ and C3H/HePas). Also, the changes on levels of chemokines can be related to the resistance to infection by *Leptospira*, but it was independent of CCL2 treatment. Association between both events suggests that the efficiency of phagocytosis can be an important factor for the resistance of a mice strain to leptospirosis. Other finding was the reduction of chemokine in the first hours after infection, which can be related to a mechanism of preventing an exacerbated immune response. On the other side, this event could favor the dissemination of bacteria in vivo infection. As shown by data analyses, CXCL5 and CCL8 interact with ACKR decoy receptors family, thus participating in the pathway cascades repressing the expression of important molecules (Fig. 4 and Additional file 1: Table S2) that could open a way for migration of leptospires in the blood of the host.

We observed that leptospires bind to the surface of macrophages, even after the antibiotic treatment. Phagocytosis involves a series of coordinated events, starting by bacterial attachment to host cell surfaces followed by activation of cell signaling pathways that lead to bacterial uptake and destruction. However, pathogenic *Leptospira* are able to survive and replicate in the macrophages, allowing their eventual spread to target organs [40]. In Figs. 2 and 3, we can observe leptospires attached to the macrophage membrane surface, corroborating the data from Toma and collaborators [40] that reported leptospires binding to the membrane and internalized in the cell after 1 h.

In our assays we detected a significant increase of CCL9 in Raw 264.7 cells when compared to control. These data are in accordance with results from Ravindran and collaborators [41], showing a potential involvement of CCL9 and its CCR1 receptor in macrophages regulation.

Furthermore, cells from the different mouse strains had different patterns of expression when submitted to these stimuli, suggesting that it could be related to their different susceptibility/resistance phenotype. Indeed, the inhibition of expression of CXCL5 and CCL8 detected in C3H/HeJ had been already detected by our group in other conditions [28], which gives further support to the idea that the differences in the control of chemokines expression may be strongly related to the susceptibility/resistance phenotype. This chemokine was not detected in vitro in cells from susceptible mouse strains C3H/HeJ and C3H/HePas in our experimental conditions, and it was inhibited in BALB/c mice spleen cells. Data analyses [42] demonstrate that CCL8 and CXCL5 chemokines are strongly related to the defense processes, including T cell chemotaxis (Additional file 2: Table S1). Our results emphasize that the production of these chemokines have important function in specific organs like lungs in the control of leptospirosis disease as reported before [28].

Interestingly, these differences in cytokine modulation did not alter the phagocytosis levels, suggesting that the signaling promoted by these modulations does not significantly change phagocytosis levels, and probably are correlated with the mechanisms of control of the host in vivo.

The concept that some bacterial pathogens evade intracellular defenses by releasing proteins that triggers the destruction of members of a key family of host enzymes is emerging [43]. Proost et al. [14] have demonstrated that the proteolytic cleavage of CCL8 converts this molecule into a potent inhibitor of chemokine-induced chemotaxis.

Other important aspect highlighted in recent studies is that some pathogenic *Leptospira* can evade intracellular defenses and even survive inside the host cells [40, 44, 45]. It is described in the literature that host proteases may regulate immune responses via cleavage of many cytokines and chemokines. This regulation is involved in immune and physiological functions, such as angiogenesis. Decreasing in functional potency of these immunes mediators was described for CCL3/MIP-1 and CCL5/RANTES [46] and for CCL8 [15].

In the present in vitro evaluation, we have detected a decrease of CXCL5 chemokine in all spleen cell lines studied, under different conditions after infection with *L. interrogans*, suggesting that the CCL2 pre-treatment and infection has potential inhibiting action of CXCL5 expression in the spleen cells, differently from lungs from in vivo infection. In previous studies, our group has shown a significant increase of CXCL5 in lung and spleen of BALB/c mice after 1d of the infection with *Leptospira* [28]. These findings indicate that the production of CXCL5 is tissue-specific and show a relevant role in controlling infection in vivo, mainly in leptospirosis-affected lungs. CXCL5 is a chemoattractant chemokine from CXC family that activates neutrophils and also plays a role in neutrophil trafficking during lung inflammation induced by LPS [17].

In fact, in vivo studies demonstrated an immediate inhibition of CXCL5 expression in susceptible mouse strain and elevation of this chemokine in the resistant strain after 24 h after of infection, indicating that a regulatory process occurs immediately after infection and that the inhibition process is maybe related to the susceptibility to infection in leptospirosis diseases [28].

We observed that exposure of spleen cells from C3H/HePas to CCL2 interferes on CCL5 content in such a manner that it was not even detected. CCL5 was not detected in C3H/HeJ exposed or not to CCL2. On the other hand, CCL5 content was not modified in BALB/c cells, illustrating the complex mechanism of modulation among the chemokines. Other examples of immune modulations via cytokines and chemokines content were described in the literature, such as the action of proteases decreasing the functional potency of the CCL3/MIP-1 and CCL5/RANTES [46] and in CCL8 [15]. Besides, data analyses network indicate that different interactions could modulate the actions of the studied chemokines. Chemokines gradients are established and regulated via complex mechanisms, including proteolytic process and receptor binding scavenging [17, 47, 48].

BALB/c that presents the TLR2 and TLR4 on the cell surface, recognizes antigens on the surface of the pathogens, thus activating the immunological processes and the consequent control of the infection, either by direct control of leptospires invasion or controlling the exacerbated production of chemokines and correlated molecules. In this context, C3H/HeJ mouse that lack TLR4 are not able to express some chemokines, which might be related to its susceptibility. Hence, these results indicate that the distinct chemokine profiles may be related to the different outcomes in chronic and acute leptospiral infections.

Dey and collaborators have demonstrated that chemokine treatment restored capacity of antigen presentation of the infected macrophages, suggesting that the chemokines are giving protection not only via free-radical generation, but they are also involved in the induction of Th1 immune response in leishmaniosis [49]. It is possible that these events are occurring also in leptospirosis.

## Conclusions

An important finding in this work was that cells from BALB/c, the resistant strain, presented the highest number of leptospires, followed by C3H/HePas and C3H/HeJ, the most susceptible strain, indicating a correlation between the number of phagocytosed *Leptospira* and the level of resistance to the infection.

CCL2 did not affect their phagocytosis efficiency, but did modulate other chemokines expression during *Leptospira* infection. BALB/c that presents TLR4+ has a greater ability to early modulation of chemokines expression, suggesting a relationship between resistance mechanisms and early modulation of chemokines in leptospirosis diseases.

Besides the correlation on phagocytosis, chemokine modulation and resistance to leptospire infection detected in this work, other studies are needed to precise the strategies involved in the control of the colonization.

## Additional files

Additional file 1: **Table S2.** Protein interactions network analysed by GeneCard-GPSProt interaction database. (XLSX 15 kb)

Additional file 2: **Table S1.** Protein interaction network analysed by String database. (XLSX 35 kb)

## Abbreviations

ACK: Ammonium-Chloride-Potassium
ACKR: atypical chemokine receptor
CCL: chemokine ligand family CC
CCL12/MCP5 C: C motif chemokine 12/monocyte chemotactic protein 5
CCL2/MCP1 C: C motif chemokine ligand 2/monocyte chemoattractant protein-1
CCL5/RANTES C: C motif chemokine ligand 5
CCL8/MCP2 C: C motif chemokine ligand 8/monocyte chemotactic protein 2
CCL9/MIP-1γ C: C motif chemokine ligand 9/macrophage inflammatory protein 1-gamma
CCR2 C: C motif chemokine receptor 2
CXCL: chemokine ligand family CXC
CXCL10/IP-10: C-X-C motif chemokine ligand 10
CXCL12/SDF-1: C-X-C motif chemokine ligand 12/stromal cell-derived factor 1
CXCL5/ENA-78: C-X-C motif chemokine ligand 5/epithelial neutrophil-activating peptide 78
DAPI 4′,6: Diamidine-2′-phenylindole dihydrochloride
LPS: lipopolysaccharide
MOI: multiplicity of infection
qPCR: quantitative PCR
